# DEVELOPING COMPETENCE IN QUALITY IMPROVEMENT: A MODEL FOR GEROPSYCHOLOGY TRAINING

**DOI:** 10.1093/geroni/igz038.2225

**Published:** 2019-11-08

**Authors:** Lindsey Jacobs, Michelle Mlinac

**Affiliations:** 1 VA Boston Healthcare System, Brockton, Massachusetts, United States; 2 VA Boston Healthcare System, Boston, Massachusetts, United States

## Abstract

Quality improvement (QI) efforts are imperative to ensuring patient-centered, safe, effective, timely, efficient, and equitable healthcare. QI training is well-established in the fields of geriatric medicine and nursing but is lacking in geropsychology. Clinical geropsychologists are in need of QI knowledge and skills, as the population they serve is often vulnerable to healthcare disparities due to complex medical, neurological, and/or psychiatric presentations, limited resources, and low social and/or instrumental support. This presentation will describe a developmental model of QI training, driven by initial pilot data from geropsychology trainees, analysis of geropsychology graduate program marketing materials, and expert opinion from geropsychologists who have QI implementation experience. Consistent with the Pikes Peak Competencies for Geropsychology Training, this model delineates aspirational QI knowledge and skills, as well as recommendations for incorporating QI training into geropsychology at progressing levels of competency from graduate school through independent clinical practice.

